# 7-Nitroindazole reduces L-DOPA-induced dyskinesias in non-human Parkinsonian primate

**DOI:** 10.1098/rsob.220370

**Published:** 2023-05-17

**Authors:** M. T. Herrero, J. E. Yuste, L. Cuenca-Bermejo, P. Almela, L. Arenas-Betancur, V. De Pablos, A. Gonzalez-Cuello, E. Del Bel, J. Navarro-Zaragoza, E. Fernández-Villalba

**Affiliations:** ^1^ Clinical and Experimental Neuroscience (NiCE), Institute for Aging Research, School of Medicine, Campus Mare Nostrum, The European University for Well-Being, EUniWell, University of Murcia, Spain; ^2^ Department of Pharmacology, School of Medicine, University of Murcia, Campus Mare Nostrum, 30100 Murcia, Spain; ^3^ Biomedical Research Institute of Murcia (IMIB-Pascual Parrilla), Campus of Health Sciences, University of Murcia, 30120 Murcia, Spain; ^4^ Department of Basic and Oral Biology, Faculty of Odontology of Ribeirão Preto (FORP-USP) and Center for Research Support on Applied Neuroscience (NAPNA-USP), University of São Paulo, Ribeirão Preto, SP 14040-904, Brazil

**Keywords:** Parkinson's disease, NO, 7-NI, dyskinesias, L-DOPA

## Abstract

Nitric oxide (NO) plays a pivotal role in integrating dopamine transmission in the basal ganglia and has been implicated in the pathogenesis of Parkinson disease (PD). The objective of this study was to ascertain whether the NO synthase inhibitor, 7-nitroindazole (7-NI), is able to reduce L-DOPA-induced dyskinesias (LIDs) in a non-human primate model of PD chronically intoxicated with 1-methyl-4-phenyl-1,2,3,6-tetrahydropyridine (MPTP). Six Parkinsonian macaques were treated daily with L-DOPA for 3–4 months until they developed LIDs. Three animals were then co-treated with a single dose of 7-NI administered 45 min before each L-DOPA treatment. Dyskinetic MPTP-treated monkeys showed a significant decrease in LIDs compared with their scores without 7-NI treatment (*p* < 0.05). The anti-Parkinsonian effect of L-DOPA was similar in all three monkeys with and without 7-NI co-treatment. This improvement was significant with respect to the intensity and duration of LIDs while the beneficial effect of L-DOPA treatment was maintained and could represent a promising therapy to improve the quality of life of PD patients.

## Graphical abstract

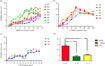

## 1. Introduction

While L-DOPA is the most effective treatment for Parkinson's disease (PD), its chronic administration leads to motor complications expressed as fluctuations in clinical responses and the appearance of abnormal involuntary movements (AIMs), known as L-DOPA-induced dyskinesias (LIDs) [1]. Effective treatment for LIDs is limited because, although molecular-based research has suggested a potential role for several neurotransmitters and receptors [2–4], the mechanisms underlying this phenomenon are still unclear [5–7]. Dopaminergic stimulation of the denervated striatum is a key mechanism underlying LIDs [8]. It can exacerbate the sensitization process [9], induce activation pathways that provoke post-synaptic plastic changes in basal ganglia circuits and facilitate AIMs [10], probably due to corticostriatal dendritic spine alterations [11].

Besides dopaminergic mechanisms, alterations in several non-dopaminergic systems have been linked to LIDs pathophysiology [12,13]. Significantly enhanced glutamatergic neurotransmission has been found in the basal ganglial-thalamo-cortical circuit [14,15] and excessive amounts of N-methyl-D-aspartate (NMDA) receptor are present in the striatum of Parkinsonian patients and animals with LIDs [16]. Thus, amantadine, non-competitive antagonist of NMDA receptor, has been used to the treatment of LIDs in Parkinson's disease patients [17,18]. However, it has been shown that this drug produces undesirable side effects in experimental and clinical models [18–20].

A growing body of evidence suggests that nitric oxide (NO) plays a role in the maintenance of LIDs [21–26], since it is synthesized in striatal interneurons by NO synthase (NOS) [27–29]. NO modulates the striatal function, changing its input–output relationship and producing a functionally significant impact on target neurons [30–33]. It has been suggested that (i) NO synthesis increases in the basal ganglia in experimental parkinsonism and in chronic treatment with L-DOPA [34–37], and (ii) nitrate concentrations and nitrite levels are high in the cerebrospinal fluid of patients with Parkinson's disease receiving dopamine replacement [38] and in patients with PD and LIDs [39]. Importantly, a potent NOS inhibitor, 7-nitroindazole (7-NI), has been successfully tested in dyskinetic rodents showing reduced AIMs [21–25,32,40–46].

Despite the large number of studies carried out in rodents, as far as we know there are no studies performed in non-human primates. Although the study in rodents is relevant, it must be taken into account that dyskinesias in non-human primates are remarkably similar to those seen in humans and the genetic and anatomo-physiological similarities with humans are greater than those of other rodent models such as rats and mice. Therefore, our aim was to determine whether the administration of 7-NI could be an efficient and safe treatment for reducing LIDs in non-human primates without affecting the therapeutic effect of L-DOPA.

## 2. Materials and methods

### 2.1. Non-human primate model of Parkinson disease

Monkeys were supervised by veterinarians and technicians skilled in the healthcare and maintenance of non-human primates. The animals were housed in primate cages under controlled conditions of humidity, light and temperature, and food (Masuri primate diet; Scientific Dietary Services, UK), fresh fruit and water were available *ad libitum*. Experiments were performed with six adult male cynomolgus monkeys (*Macaca fascicularis*, 3.8–4.5 kg) (purchased from R.C. Hartelust BV, The Netherlands) that were rendered Parkinsonian with methyl-4-phenyl-1,2,3,6-tetrahydropyridine (MPTP)-hydrochloride (Sigma, 0.3-0-4 mg kg^−1^ i.v. for a maximum period of 6 months, one injection every 2 weeks, *n* = 6) as previously described [47] (table 1). After reaching a stable parkinsonism (SP), monkeys were treated daily with Madopar [48] (Roche, 100 mg kg^−1^ L-DOPA and 25 mg kg^−1^ benserazide; ratio 4 : 1, *n* = 6) (termed L-DOPA hereafter) for 4 months until they developed stable and moderate–severe LIDs. 25 mg kg^−1^ of the NOS inhibitor (7-NI; Sigma-Aldrich) was dissolved in peanut oil solution and was administered subcutaneously every day 45 min prior to the injection of L-DOPA for 25 additional days (*n* = 3, randomly chosen).

**Table 1.** Experimental details of MPTP intoxication. Number and total dose of MPTP injections (in mg) and disability score before MPTP intoxication.

monkey	MPTP injections (number)	total dose (mg of MPTP)	disability score before
1	15	16.15	0
2	15	18.74	0
3	15	20.29	0
4	15	26.02	0
5	15	28.55	0
6	15	23.37	0

### 2.2. Behavioural assessments and clinical scales

The level of parkinsonism was assessed with a previously described [49,50] motor scale which evaluates the following symptoms: akinesia/bradykinesia, freezing, tremor, self-feeding, posture and spontaneous activity (maximum disability score, 25). Parkinsonian disability was assessed at the end of each session so as not to interfere with the assessment of levels of general activity. All monkeys reached similar SP levels. The intensity of LIDs was rated for each body segment (face, neck, trunk, arms and legs) every 30 min using a Dyskinesia Disability Scale (maximal score of 21 points) [51]. The dyskinetic score obtained was the sum of the scores for all body segments. LIDs were mainly choreic but dystonia was also observed. Stereotypies or licking were not considered as LIDs. The animals were placed in special observation cages for filming and the dyskinetic score was evaluated blindly with and without 7-NI co-administration. Motor evaluation was performed in the following endpoints: MPTP group when the six months of intoxication were finished, MPTP + L-DOPA group when the four months of treatment were finished and the group MPTP + L-DOPA + 7NI when the 25 days of 7NI treatment was completed.

### 2.3. Statistical analysis

Comparisons were performed using one-factor ANOVA with repeated measures followed by Newman–Keuls *post hoc* analysis. The area under the curve (AUC) [52], derived from the time-course experiments, was analysed by unpaired *t*-test. All data were expressed as mean group value ± s.e.m. A probability level of 5% (*p* < 0.05) or 1% (*p* < 0.01) was considered significant or very significant, respectively.

## 3. Results

**Figure 1.**
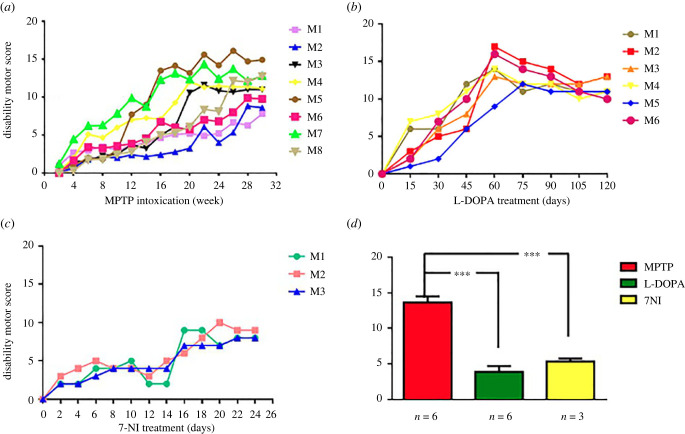
Disability motor score values in three different pharmacological states: (*a*) prior to L-DOPA treatment SP, (*b*) after L-DOPA treatment and (*c*) after L-DOPA + 7-NI co-administration. (*d*) Graph shows significant differences in disability motor score between SP data treated with MPTP compared with values observed after treatment of Parkinsonian mice with DOPA or L-DOPA + 7-NI co-administration; data are represented as mean ± s.e.m. *p* < 0.001 versus SP.

**Figure 2.**
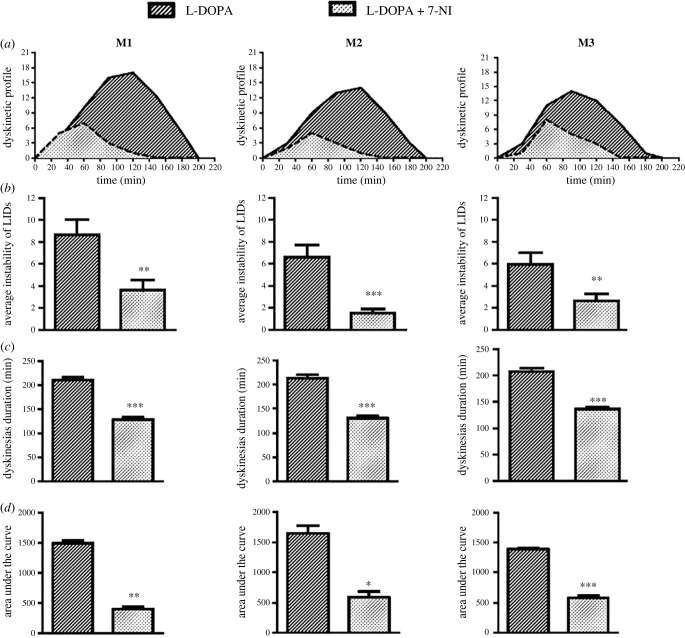
(*a*) Dyskinetic profile showing the intensity (dyskinesias score) and duration (min) in the three monkeys (M1, M2 and M3) with L-DOPA with and without 7-NI co-administration. (*b*) 7-NI significantly reduces the average intensity of LIDs over the time course: M1 without (9/21) and with (4/21) 7-NI co-treatment, M2 without (7/21) and with (1/21) 7-NI co-treatment, and M3 without (6/21) and with (3/21) 7-NI co-treatment (*t*-test, ** *p* < 0.01 and ***p < 0.001); and (*c*) duration of LIDs in MPTP-treated monkeys: M1 without (200 min) and with (130 min) 7-NI co-treatment, M2 without (200 min) and with (135 min) 7-NI co-treatment, and M3 without (200 min) and with (140 min) 7-NI co-treatment (*t*-test, ***p < 0.001). (*d*) The AUC, a measure that encompasses both intensity and duration of the LIDs, was significantly reduced with 7-NI co-administration for each monkey (paired *t*-test, ***p < 0.001 L-DOPA + 7-NI versus L-DOPA without 7-NI). These data are expressed as the mean AUC ± s.e.m. between 0 and 200 min in the three monkeys.

We assessed the ability of 7-NI to change L-DOPA-induced diskinesia. Our study demonstrated that MPTP-treated monkeys developed moderate–severe LIDs when they are treated for 4 months with L-DOPA. The anti-Parkinsonian effect of L-DOPA was similar in all six monkeys, whose disability motor scores compared with their stable Parkinsonian state improved significantly (figure 1*a,b,d*). Their dyskinetic profiles showed a similar maximum (peak dose) at 80–100 min, finishing at 190–200 min. 7-NI co-administration preserved the beneficial anti-Parkinsonian effect of L-DOPA treatment without significant differences in the disability motor score (*p* > 0.05) (figure 1*c,d*). 7-NI dramatically decreased the intensity (figure 2*b*) and duration (figure 2*c*) of the LIDs, reducing the profile by more than 50%. Analyses of the time course and overall dyskinetic response (AUC) showed that 7-NI significantly reduced LIDs (*p* < 0.001) (figure 2*d*).

*Monkey 1* received nine injections of MPTP over a period of four and half months. It developed significant bradykinesia, rigidity and freezing after the third MPTP injection. After the fifth MPTP injection resting tremor in upper limbs also appeared, and it showed a transient dystonia in the inferior limbs and in the oromandibular area, which disappeared after the eighth MPTP injection. After four months of L-DOPA treatment, stable body trunk LIDs, stereotypies in the hands, jerking motions and dystonic postures of lower limbs and tail developed. Fast choreic movements appeared 10 min after L-DOPA administration, lasting 200 min and reaching the peak dose at 110–120 min (intensity of 17/21). From the first co-administration with 7-NI, the dyskinetic profile changed, becoming significantly shorter (finishing at 130 min) (*p* < 0.001) (figure 2*a*,*c*) and reaching the peak dose at 60 min with a score of 6/21 (*p* < 0.001) (figure 2*b*,*c*).

*Monkey 2* received nine injections of MPTP over four and half months. It developed rigidity, severe bradykinesia and slight tremor in upper limbs, and occasionally in the head. On several occasions following MPTP injection, abnormal movements of the mouth (repeated chewing) were observed. After four months of L-DOPA treatment, stable choreic movements of the whole body, but especially in the lower limbs appeared 10–15 min after L-DOPA administration lasting 200 min and reaching the highest peak dose at 120 min (intensity of 14/21) (figure 2*a*). From the first co-administration with 7-NI, the duration of LIDs was significantly shorter (finishing at 135 min) (*p* < 0.001) (figure 2*a*,*c*), reaching the peak dose at 60 min with a maximal score of 5/21 (*p* < 0.001) (figure 2*b,c*).

*Monkey 3* received 15 injections of MPTP during a period of six months. Some vegetative symptoms were observed immediately after each dose but the animal returned to a normal state within 24 h. It showed rotational behaviour after the fourth MPTP injection. Bradykinesia, rigidity, freezing and tremor in the upper limbs, and oromandibular dystonia appeared after the seventh dose of MPTP. After four months of L-DOPA treatment stable choreic movements of all the body, especially fast in the lower limbs with the toe twisted, appeared 10–15 min after L-DOPA administration, lasting until 200 min and reaching the highest peak dose at 90 min (intensity of 14/21, figure 2*a*). From the first co-administration with 7-NI, the duration of LIDs was significantly shorter (finishing at 140 min) (*p* < 0.001) (figure 2*a,c*) reaching the peak dose at 60 min with a maximal score of 8/21 (*p* < 0.001) (figure 2*b,c*).

## 4. Discussion

Based on clinical data showing a direct correlation between the risk of LIDs development and the total amount of L-DOPA intake, a reduction in the daily doses of L-DOPA has been proposed by combining low doses of L-DOPA with non-dopaminergic therapies (for review, see [53]). In the present study, 7-NI, a NOS inhibitor, potently reversed LIDs in monkeys. The antidyskinetic efficacy was not accompanied by detrimental effects on Parkinsonian motor symptoms. 7-NI effectively reduced the LIDs score but did not affect the disability motor score in MPTP-treated monkeys and injected with L-DOPA. Previous studies have demonstrated that the NOS inhibitor, 7-NI attenuated LIDs in 6-hidroxydopamine (6-OHDA) rats [21–25,32,40–43,45,46]. However, the present study provides the first evidence that 7-NI is effective against LIDs in MPTP-treated monkeys, which have been recognized as one of the best animal models to predict the clinical efficacy of compounds on LIDs (see [54,55] for a review).

Various measures of striatal NOS activity have indicated that NO signalling may be disrupted in patients with PD [56,57] and dopamine-depleted rats [58,59]. Studies by Del Bel *et al*. [21] in animals with an intact dopaminergic system, striatal NO—the enzyme soluble guanilate-cyclase (sGC)—cyclicGM Phosphate transmission is likely to play a role in facilitating locomotor activity. Intrastriatal exposure to NOS and sGC inhibitors has been shown to depress basal locomotion and induce catalepsy [21,60]. However, we observed no cataleptic or lethargic effects in a motor evaluation of the monkeys. In fact, previous studies performed in baboons showed that 7-NI administration causes hyperactivity [61]. We observed no evidence of this in the 7-NI-treated-animals. Analysis of the AUC, which encompasses both the intensity and duration of the total dyskinetic response (figure 2*d*), demonstrates that the inhibition of NOS constantly and significantly reduces the LIDs profile (figure 2*b,c*). Pre-clinical studies aimed at decreasing NO signalling (and cGMP levels) have shown that co-administration of NOS inhibitors with L-DOPA attenuates LIDs [22,62]. NOS inhibition also improved the motor performance of the same animals on a rotarod test [22].

A possible mechanism to explain this phenomenon could be the role of striatal nitrergic interneurons, which are activated by both corticostriatal synaptic transmission (by direct synaptic contacts) and dopaminergic terminals (by D1/5 receptors) within striatal neural networks [33,63]. In fact, striatal NO plays a critical role in (i) activating cGMP in the medium spiny neurons (MSNs) upregulating cAMP [64] and inhibiting glutamate release in corticostriatal pathways [65], and/or (ii) provoking long-term depression in MSNs [33,66]. Therefore, we think it investigating the role of nitrergic pathways in LIDs using Parkinsonian monkeys would be of interest in future research.

Supported by many studies it has been proposed that abnormal neurotransmission and pathways not only dopamine may be involved in the pathophysiology of LIDs. Thus, recent paper has shown that a selective inhibitor of phosphodiesterase 1(PDE1) has potent antidyskinesic efficacy in non-human primate [67]. Further, several NMDA antagonists have shown efficacy against LIDs in chronically MPTP-treated monkeys [68], so that amantadine has been approved as treatment of LIDs [69]. However, amantadine has several adverse effects including hallucinations, constipation, dry mouth, peripheral oedema and nausea [70].

## 5. Opening up

There is interest to find new antidyskinesic candidates with the better safety profiles than amantadine. In this context, glutamate receptors are described to interact with NO, which may inhibit NMDA receptor by protein nitrosylation and destabilization of synaptic proteins [71]. Moreover, since 7-NI is able to reduce LIDs in Parkinsonian monkeys, different approaches could be evaluated using 7-NI co-administration. Many new selective neuronal NOS inhibitors are accessible, with reduced off-target effects related to other isoforms and promising pharmacokinetics to access brain tissues [72]. Additionally, Titze-de-Almeida *et al*. [73] described a siRNA which triggered a knockdown of nNOS mRNA and protein, reducing the cytotoxicity caused by 6-OHDA on SH-SY5Y cells. The intra-striatal injection of the siRNA-protected nigral dopaminergic neurons in 6-OHDA hemi-lesioned rats. Further on, in rodents 7-NI decreases dopamine turnover, (DOPAC/dopamine ratio) in the striatum of dyskinetic rats, which suggests the increase in the dopamine availability [41]*.* Ultimately, the combined treatment of amantadine and the NO inhibitor (7-NI), both in low doses, may result in fewer side effects and a superior therapeutic benefit in LIDs [45].

## 6. Conclusion

Our results demonstrated that 7-NI is able to reduce the intensity and duration of LIDs without affecting anti-Parkinsonian benefits and could represent a promising therapy to improve the quality of life of PD patients.

## Data Availability

Original data are available upon request to mtherrer@um.es.
